# Retarded Congenital Syphilis

**Published:** 1867-08

**Authors:** H. Durham

**Affiliations:** LaSalle, Ill.


### RETARDED CONGENITAL SYPHILIS.—SYPHILITIC CARIES.

By H. Durham, M.D., LaSalle, Ill.

Case. H. B., aged 8 years, 4 ft. 2 in. in height, with light, almost sallow, complexion and hair, sanguino-bilious temperament, with no previous sickness, eruption, or sores; of a syphilitic father, cured twenty years ago in a military hospital in France, and of a scrofulous mother, to whom the disease was not communicated by husband or child, and whose two previous children (the first of the two by a former husband) have shown no specific symptoms, last June complained of pain in his left foot, when, in a day or two, a sore appeared upon the tarsal integument, that soon ulcerated with great rapidity, with well-defined edges and circumscribed redness and inflammation.

On the third day after the appearance of the ulcer, Dr. Bry, the attending physician, who had diagnosed the case, called me in, when the cuboid and external and middle cuneiform bones, with the metatarsal bones of the 4th and 5th toes were denuded, and nearly all of them were ready to drop, and, with the exception of the cuneiform bones, were removed with the phalanges. The child was treated with the proto-iodide of mercury three days, and then with iodide of patass. and compound syrup of sarsaparilla, and made a rapid recovery.

The annals of science furnish few well-authenticated cases of syphilitic lesions of the bones at this early age, and they seldom assume the well-known forms that make their diagnosis so easy in later years. Bertin speaks of having seen periostitis. Laborie quotes a case of caries of tibia. Cruvelhier mentions caries of the orbital vault. Rosen refers to a case of the carious hard palate. But probably some of these were cases of necrosis from ulcerative denudation.

*Teachings of the case.*—1st. Syphilis is propagated otherwise than from a chancre solely. This child never had a chancre.

2d. The lesion was syphilitic. The symptoms were well marked, and specific treatment altered the character of the ulceration in three days.

3d. The father, at the time of fecundation, had secondary syphilis, and was in a condition to transmit the disease.

4th. The mother did not contract the disease either from the husband or from the embryo *in uterus.* Her health has not declined since marriage.

5th. The child never had a syphilitic symptom previously. At the sitting of the Academy of Medicine, October 8th, 1853, Ricord says, he had a case, aged 17, who inherited tertiary syphilis from his father, and had seen subjects in whom hereditary syphilis did not *manifest* itself before the age of 40.

6th. The different stages, as such, are not transmissible. The father had secondary syphilis, and the child showed tertiary symptoms; but, in truth, the symptoms of congenital syphilis lie between the two stages, partaking of both.

7th. The power of treatment becomes exhausted. The girl of the mother by a former marriage, aged 14, shows no symptoms; the elder boy, aged 10, by this marriage, shows none; the patient, the younger boy, aged 8, does give syphilitic manifestations. How shall we account for it, but under the supposition that at the time of the first procreation, the father was still under the influence of mercury, and that by the time of the second procreation the specific influence of mercury on the system had been lost! This seems more probable, from the fact that the mother has since miscarried once.

Diday says, a father may beget a syphilitic child, though no symptoms may appear in 10 years. Albers gives his opinion, that retarded syphilis chiefly affects children of a syphilitic father and a scrofulous mother, which opinion the present case confirms.

8th. Venereal dyscrasis may attack one who has never shown debility; contrary to Diday's teaching, who holds that the inherited disorder always brings a debility which predisposes the constitution to acute diseases, etc. Troncin teaches that if the syphilitic child do not die at birth, it will have glandular swellings, a large and tympanitic abdomen, and tardy dentition, none of which conditions are fulfilled in the case of H. B. He also thinks that the slightest disease will carry it off, while Sperino saw a child that, after two *years* of sickness, the disease had perforated the hard palate by ulceration!

9th. The disease chooses the weakest spot for an outbreak. But why should that be in the foot? We could understand it, if the child had received a severe blow or a sprain to occasion congestion, which might cause ulceration in syphilitic subjects, just as congestion produces ulceration of the bowels and induration of the liver in like sufferers. But the child had received no injury, and we must account for the location of the ulceration from the dependent condition of the part, the distance from the centre of circulation, the frequent use of the foot in the restlessness of childhood, and the consequent rapid growth of the bones.

10th. Though congenital syphilis commonly proves fatal before it reaches the tertiary stage, it may first appear at that very stage, as this child never had the least indication of a syphilitic diathesis previous to this attack.

